# Anorexia nervosa: 30-year outcome

**DOI:** 10.1192/bjp.2019.113

**Published:** 2020-02

**Authors:** Sandra Rydberg Dobrescu, Lisa Dinkler, Carina Gillberg, Maria Råstam, Christopher Gillberg, Elisabet Wentz

**Affiliations:** 1 Student, Gillberg Neuropsychiatry Centre, Institute of Neuroscience and Physiology, Sahlgrenska Academy, University of Gothenburg, Sweden; 2 Associate Professor, Gillberg Neuropsychiatry Centre, Institute of Neuroscience and Physiology, Sahlgrenska Academy, University of Gothenburg, Sweden; 3 Professor of Child and Adolescent Psychiatry, Department of Clinical Sciences Lund, Lund University; and Visiting Professor, Gillberg Neuropsychiatry Centre, Institute of Neuroscience and Physiology, Sahlgrenska Academy, University of Gothenburg, Sweden; 4 Professor of Child and Adolescent Psychiatry, Gillberg Neuropsychiatry Centre, Institute of Neuroscience and Physiology, Sahlgrenska Academy, University of Gothenburg, Sweden; 5 Professor of Psychiatry, Department of Psychiatry and Neurochemistry, Institute of Neuroscience and Physiology, Sahlgrenska Academy, University of Gothenburg, Sweden

**Keywords:** Anorexia nervosa, outcome, population based, case–control

## Abstract

**Background:**

Little is known about the long-term outcome of anorexia nervosa.

**Aims:**

To study the 30-year outcome of adolescent-onset anorexia nervosa.

**Method:**

All 4291 individuals born in 1970 and attending eighth grade in 1985 in Gothenburg, Sweden were screened for anorexia nervosa. A total of 24 individuals (age cohort for anorexia nervosa) were pooled with 27 individuals with anorexia nervosa (identified through community screening) who were born in 1969 and 1971–1974. The 51 individuals with anorexia nervosa and 51 school- and gender-matched controls were followed prospectively and examined at mean ages of 16, 21, 24, 32 and 44. Psychiatric disorders, health-related quality of life and general outcome were assessed.

**Results:**

At the 30-year follow-up 96% of participants agreed to participate. There was no mortality. Of the participants, 19% had an eating disorder diagnosis (6% anorexia nervosa, 2% binge-eating disorder, 11% other specified feeding or eating disorder); 38% had other psychiatric diagnoses; and 64% had full eating disorder symptom recovery, i.e. free of all eating disorder criteria for 6 consecutive months. During the elapsed 30 years, participants had an eating disorder for 10 years, on average, and 23% did not receive psychiatric treatment. Good outcome was predicted by later age at onset among individuals with adolescent-onset anorexia nervosa and premorbid perfectionism.

**Conclusions:**

This long-term follow-up study reflects the course of adolescent-onset anorexia nervosa and has shown a favourable outcome regarding mortality and full symptom recovery. However, one in five had a chronic eating disorder.

Follow-up studies of anorexia nervosa have been conducted since the second half of the 20th century and were thoroughly reviewed in 2009 by Steinhausen.^1^ According to the review, approximately half of all individuals with anorexia nervosa were classified as fully recovered, one in three had improved and one in five had a chronic course of the disorder. The crude mortality rate was 5%.^1^ High mortality rates were also found in a meta-analysis reporting a standardised mortality ratio of six for anorexia nervosa.^2^ According to Steinhausen, the outcome of adolescent-onset anorexia nervosa was more favourable in terms of mortality and chronicity than the outcome of anorexia nervosa with variable onset, including patients with adult onset.^1^ All 119 patient series in the Steinhausen review^1^ except two – the FinnTwin study^3^ and the present study, i.e. the ‘Gothenburg anorexia nervosa study’,^4–7^ – were based on clinical data.

## Studies with exceptionally long observational periods

Three anorexia nervosa outcome studies (one from Germany,^8^ one from the USA^9^ and one from Sweden^10^) report exceptionally long observational periods of more than 20 years. All three studies included patients only. Recovery from anorexia nervosa was observed in 51% of patients after 21 years in the German study,^8^ in 63% of patients after 22 years in the American study^9^ and in 76% of patients after 33 years in the Swedish study.^10^ Two of the studies reported crude mortality rate, corresponding to 16 and 18%, respectively.^8,10^ The three outcome studies either performed personal interviews^8,10^ or telephone interviews^9^ at follow-up. Two of the studies had a prospective design.^8,9^

Since 1985, we have carried out a prospective, longitudinal, case–control study of individuals with adolescent-onset anorexia nervosa. The individuals have been examined on four previous occasions. The aims of the present study were as follows:

- to prospectively examine the very long-term outcomes of adolescent-onset anorexia nervosa, including full recovery from eating disorder symptoms, psychiatric morbidity, mortality, global functioning and health-related quality of life (HRQoL).
- to identify predictors of outcome to determine risk factors for developing anorexia nervosa.

For our study group we hypothesised that the outcomes of Global Assessment of Functioning (GAF), HRQoL, eating disorder outcome and psychiatric morbidity would be significantly worse than for the matched comparison group. We hypothesised that the outcomes within our study group would be better than the outcomes of the clinical, long-term studies due to this sample being partly community and population based and only including individuals with adolescent-onset anorexia nervosa.

## Method

### Study design and participants

#### The original study (study 1)

##### The anorexia nervosa group

In 1985, the so-called Gothenburg anorexia nervosa study was initiated by M.R. and C.G. All 4291 individuals born in 1970 and attending eighth grade in Gothenburg underwent a physical examination and completed an eating disorder symptom questionnaire. All 4291 growth charts and questionnaires were scrutinised. All individuals suspected to have anorexia nervosa were examined in person. All children in special schools were assessed at the same time. Regarding all boarding schools, M.R. established contact with the school nurses and interviewed them regarding any child born in 1970 who could have anorexia nervosa. There were no cases of home education. In the 1970 birth cohort, 23 girls and 2 boys met criteria for anorexia nervosa before age 18. Only 1 girl declined further examination, leaving 24 individuals (22 girls, 2 boys) in the ‘population-based group’ (for further details see^7^). Three individuals from the population-based group fulfilled almost all the criteria for anorexia nervosa according to the DSM-III-R (1987); these participants were considered as partial cases. These three individuals met all criteria for anorexia nervosa shortly after study 1 (see below). At the time of the epidemiological study, individuals with anorexia nervosa, born in 1969 and 1971–1974, were also reported to the researchers by the school health services after community screening. In all, 27 adolescents (26 girls, 1 boy) formed the ‘population-screening group’, which was not a clinical group. A third of the population-screening group had not been in touch with any paediatric, child and adolescent psychiatric or adult psychiatric service.

Due to the similar group structure, the two groups were pooled together to form the ‘anorexia nervosa group’, consisting of 51 participants with anorexia nervosa (48 girls, 3 boys).^7^ All 51 individuals had been assigned a diagnosis of anorexia nervosa by a physician specialised in psychiatry (M.R.) and all met the DSM-III-R^11^ and the DSM-IV (1994)^12^ criteria for anorexia nervosa. Therefore, all participants were found to be true cases of the disorder. The mean age at onset of anorexia nervosa was 14.3 years (range 10.0–17.2 years).

##### Comparison group

At the time of the anorexia nervosa screening, a comparison group was recruited. For each of the 51 participants with anorexia nervosa, the school health nurses selected a same-gender classmate who was closest in age to the index child. These comparison participants had no history of an eating disorder. This group also consisted of 51 individuals (48 girls, 3 boys).

At the time of the original study (study 1), all 102 individuals (51 anorexia nervosa, 51 comparison) underwent a physical and psychiatric examination. The mothers were interviewed in a semi-structured way and they completed questionnaires about the child's developmental history, psychiatric and personality traits, temperament, interests and peer relationships. The assessment of childhood perfectionism was made as a clinical judgement based on several-hours-long interviews with the parents (e.g. including a semi-structured validated interview).^13^ Information from a parental questionnaire, the child healthcare centre and the patient registers were also used.

M.R. compiled case notes for all individuals. All information that could raise suspicion that the child might have an eating disorder had been excluded in these records. C.G., who was blinded to group status, assigned psychiatric diagnoses (including autism spectrum disorder [ASD]) and personality traits (including perfectionism) based on the data from the case notes.^7^ In the anorexia nervosa group, 12 participants had developed bulimia nervosa after the onset of anorexia nervosa and a diagnosis of bulimia nervosa was met either before or at study 1. This information was based on growth charts, school nurse assessments and questionnaires (and reported at study 1).^7^

#### Study 2, study 3 and study 4

The anorexia nervosa and comparison groups were followed up 6 years after the onset of anorexia nervosa (study 2; mean age 21 years), 10 years after onset (study 3; mean age 24 years) and after 18 years (study 4; mean age 32 years). All 102 individuals agreed to participate in all follow-up studies.

#### Study 5 (the present study)

All 102 participants were traced. Four individuals in the anorexia nervosa group (two women, two men) declined participation (one man lived abroad and worked ‘12–15 h a day’, one woman was too disabled due to an anxiety disorder and one man and one woman were not comfortable taking part in the research project), leaving 47 participants (46 women, 1 man; 36 face-to-face interviews, 11 online video conferences/telephone interviews) in the anorexia nervosa group. All 51 individuals (48 women, 3 men; 42 face-to-face interviews, 9 online video conferences/telephone interviews) in the comparison group agreed to participate. In all, 98 out of 102 individuals participated in the study (drop-out rate 4%).

This study was approved by the Regional Ethical Review Board at the University of Gothenburg (398–14) and followed the World Medical Association's Declaration of Helsinki. The individuals participated voluntarily. Written informed consent was obtained from all participants.

### Procedures

The Mini-International Neuropsychiatric Interview (MINI 6.0)^14^ was used. Two of the individuals in the anorexia nervosa group only agreed to participate in a short interview and therefore the MINI was conducted to 45 out of the 47 participants in the anorexia nervosa group. The eating disorder module of the MINI was considered too brief, and therefore the eating disorder domain of the Structured Clinical Interview for DSM-IV (SCID-I)^15^ and a DSM-5 (2013) checklist for feeding and eating disorders were added. In the present study the DSM-5 criteria for feeding and eating disorders have been applied.^16^

The GAF was used to assess general outcome. The Morgan–Russell scales were used to calculate a ‘Morgan–Russell averaged scale score’, which is a composite score summarising body and weight concern, dieting, body weight, menstrual status, mental state, attitudes toward sexual relationships and menstruations, social relationships with family and friends, and employment during the past 6 months. The Morgan–Russell scales are well established and the best-validated outcome instruments in anorexia nervosa research.^17^

The 36-item Short Form Health Survey (SF-36),^18^ a generic HRQoL instrument, is the most frequently applied HRQoL measurement in eating disorders.^19^ It consists of four physical and four mental subscales. The instrument includes two composite scores: the Physical Composite Score (PCS) and the Mental Composite Score (MCS).

#### Full recovery from eating disorder symptoms

In accordance with our definition of full recovery from eating disorder symptoms in study 3 and study 4,^5,6^ informed by Strober *et al*,^20^ a fully recovered individual ‘refers to patients who have been free of all criterion symptoms of anorexia nervosa or bulimia nervosa for not less than 8 consecutive weeks’. It requires ‘the sustained absence of weight deviation, compensatory behaviours, and deviant attitudes regarding weight and shape, including weight phobia’. In our sample, we also required being free from all criteria of binge-eating disorder and that the individuals had been free from the above symptoms for a minimum of 6 months. In the present study information was obtained from the MINI, SCID-I, the DSM-5 checklist for feeding and eating disorders and the Morgan–Russell scales.

### Statistical analyses

Mainly non-parametric tests were used, due to the scale scores not being normally distributed. The chi-squared test or Fisher's exact test were used for dichotomous variables and the Mann–Whitney test for continuous variables. To assess change over time, McNemar's test and the Wilcoxon signed-rank test were used for dichotomous and continuous variables, respectively. A *P*-value of <0.05 was regarded as statistically significant. In cases of multiple comparisons, the upper limit of false significance was calculated as follows: (number of tests − number of significant tests on significance level 0.05)*0.05/(1–0.05).

#### Predictive factors

Predictive factors were chosen in accordance with the 18-year follow-up study (study 4)^6^ and focused mainly on variables measured at study 1 and on premorbid/retrospective childhood data. The following predictors were investigated for their individual relationships with continuous outcome variables (GAF, Morgan–Russell averaged scale score, PCS, MCS) and the dichotomous outcome variable of full recovery from eating disorder symptoms: age at onset of adolescent-onset anorexia nervosa and premorbid body mass index (BMI); lowest BMI ever assessed at study 2 (continuous); perinatal factors and social class (ordinal); early gastrointestinal problems; primary amenorrhoea; premorbid problems including problems with friends, major problems in the family, major life events, parental divorce, death of family member; affective disorder; overweight; obsessive–compulsive disorder; obsessive–compulsive personality traits; perfectionism; and ASD before study 1 and/or at study 2 (all dichotomous). Spearman correlations were used to examine the association of two continuous/ordinal variables, Mann–Whitney test for the association of continuous/ordinal with dichotomous variables and logistic regression for the univariate associations of predictors with full recovery of eating disorder symptoms. Variables with *P* < 0.10 in the univariate tests were included as possible predictors in the multiple stepwise regression analyses (‘backward’ procedure in SPSS).

## Results

There was no mortality. The mean ages at study 5 and the length of the follow-up periods are presented in Supplementary Table S1 available at https://doi.org/10.1192/bjp.2019.113. Anthropometric data are shown in Table 1.

**Table 1:** Comparisons between the anorexia nervosa and the comparison group regarding outcome variables and changes between baseline and study 5, and between study 4 and study 5

	Baseline					Anorexia nervosa study 4					Anorexia nervosa study 5				
	Anorexia nervosa group (*N* = 51)		Comparison group (*N* = 51)		*P*	Anorexia nervosa group (*N* = 51)		Comparison group (*N* = 51)		*P*	Anorexia nervosa group (*N* = 47)^a^		Comparison group (*N* = 51)		*P*
	Mean	s.d.	Mean	s.d.		Mean	s.d.	Mean	s.d.		Mean	s.d.	Mean	s.d.	
Morgan–Russell averaged scale score	n/a		n/a			9.74	2.08	11.02	1.17	<0.0001	9.60	1.82	10.67	1.63	0.003
GAF	n/a		n/a			65.18	17.98	82.39	12.84	<0.0001	60.72	17.23	80.20	14.78	<0.0001
Weight	49.52^b^	8.73	55.99	6.67	<0.0001	62.26	13.26	66.69	11.01	0.069	64.55	13.38	70.43	13.61	0.034
Height	164.61	6.95	167.08	7.42	0.06	166.78	6.52	169.00	7.03	0.101	166.20	5.97	169.09	6.80	0.028
BMI	18.28	2.90	20.02	1.75	<0.0001	22.36	4.55	23.41	4.21	0.23	23.36	5.00	24.72^b^	4.92	0.18

	Change: baseline to study 5						Change: study 4 to study 5					
	Anorexia nervosa group (*N* = 47)			Comparison group (*N* = 51)			Anorexia nervosa group (*N* = 47)^a^			Comparison group (*N* = 51)		
	Mean	s.d.	*P*	Mean	s.d.	*P*	Mean	s.d.	*P*	Mean	s.d.	*P*
Morgan–Russell averaged scale score^c^	n/a			n/a			−0.18	1.60	0.337	−0.35	1.12	0.062
GAF^d^	n/a			n/a			−4.19	13.67	0.065	−2.20	11.04	0.133
Weight^e^	15.63	14.16	<0.0001	14.50^b^	10.71	<0.0001	3.36	7.11	0.003	3.96^b^	6.18	<0.0001
Height^f^	1.71	3.47	<0.0001	2.01	3.89	<0.0001	−0.15	0.86	0.224	0.09	0.87	0.677
BMI^g^	5.31	5.16	<0.0001	4.69	3.87	<0.0001	1.23	2.57	0.002	1.34^b^	2.22	<0.0001

‘Baseline’ corresponds to study 1 (the original study), when the anorexia nervosa screening was performed. Average minimum BMI at the time was 14.9 kg/m^2^ (s.d. 2.6) in the anorexia nervosa group. Out of 51 individuals in the anorexia nervosa group, 12 no longer fulfilled an anorexia nervosa diagnosis at the time of study 1. Due to multiple comparisons the upper limit of false significance was calculated to be 1.2. Study 4, 18-year follow-up; study 5, 30-year follow-up/the present study; n/a, not applicable; GAF, Global Assessment of Functioning; BMI, body mass index (kg/m^2^).

a. Two out of three males did not participate.

b. Based on 50 individuals.

c. Change from study 4 to study 5, anorexia nervosa versus comparison group: *P* = 0.547.

d. Change from study 4 to study 5, anorexia nervosa versus comparison group: *P* = 0.427.

e. Change from study 4 to study 5, anorexia nervosa versus comparison group: *P* = 0.654.

f. Change from study 4 to study 5, anorexia nervosa versus comparison group: *P* = 0.173

g. Change from study 4 to study 5, anorexia nervosa versus comparison group: *P* = 0.832.

### General outcome

The outcome variables of the GAF and Morgan–Russell averaged scale score were significantly lower in the anorexia nervosa group (Table 1).

### Changes in outcome between study 4 and study 5

Weight and BMI had increased significantly in the anorexia nervosa and comparison group between study 4 and study 5 (Supplementary Table S1). The GAF and Morgan–Russell averaged scale score had not changed between the last two follow-ups in either group.

### Eating disorders at study 5

Nine individuals (19%) in the anorexia nervosa group had a current eating disorder, including three people with anorexia nervosa (two classified as being in partial remission. If DSM-IV criteria had been applied, one of the three people with anorexia nervosa would have been classified as eating disorder not otherwise specified due to regular menstruations) (Table 2). The mean duration of the first episode of anorexia nervosa (calculated from onset of anorexia nervosa) was 3.6 years (s.d. 3.0). The mean duration of all aggregated episodes of anorexia nervosa was 4.9 years (s.d. 5.1). The mean duration of all aggregated episodes of eating disorders (including anorexia nervosa) was 10.2 years (s.d. 8.1).

**Table 2:** The prevalence of psychiatric diagnoses at study 5 and the prevalence of psychiatric diagnoses between study 4 and study 5

	Current diagnoses						Diagnoses between study 4 and study 5					
	Anorexia nervosa group				Comparison group		Anorexia nervosa group				Comparison group	
	No eating disorder (*N* = 38)^a^	Eating disorder (*N* = 9)^a^	Total (*N* = 47)^b^		Total (*N* = 51)		No eating disorder (*N* = 30)	Eating disorder (*N* = 16)^a^	Total (*N* = 46)^a^		Total (*N* = 51)	
			*N*	%	*N*	%			*N*	%	*N*	%
Eating disorders		9	9**	19.1	1^c^	2		16	16***	34.8	1^c^	2
Anorexia nervosa		3	3	6.4	0			8^d^	8**	17.4	0	
Bulimia nervosa		0	0		0			0	0		0	
Binge-eating disorder		1	1	2.1	0			3	2	4.3	0	
OSFED		5	5^e^	10.6	1^c^	2		6^f^	6*^f^	13.0	1^c^	2.0
Any affective disorder	6	2	8	17.8	3	5.9	11	11	22*	48.9	13	25.5
Any anxiety disorder	8	5	13*	28.9	5^g^	9.8	9	10	19**	42.2	7	13.7^g^
OCD	4	2	6	13.3	2^g^	3.9	3	4	7	15.6	2^g^	3.9
Any psychiatric disorder excluding eating disorders	12	5	17**	37.8	6	11.8	15	11	26**	57.8	15	29.4
Any psychiatric disorder including eating disorders	12	9	21***	46	6	11.8	15	16	31***	67.4	15	29.4

For feeding and eating disorders the DSM-5 criteria have been applied; criteria for other psychiatric disorders were based on the DSM-IV (Mini-International Neuropsychiatric Interview [MINI] 6.0). Study 4, 18-year follow-up; study 5, 30-year follow-up/the present study; OSFED, other specified feeding or eating disorder; OCD, obsessive–compulsive disorder.

a. One individual was interviewed regarding eating disorders but not regarding other psychiatric disorders, i.e. no MINI was performed.

b. Two individuals were interviewed regarding eating disorders but not regarding other psychiatric disorders; i.e., no MINI was performed.

c. OSFED night eating syndrome.

d. One individual with anorexia nervosa also had binge-eating disorder between study 4 and study 5.

e. Two individuals had OSFED atypical anorexia nervosa, one individual had OSFED bulimia nervosa, one individual had OSFED binge-eating disorder and one individual had OSFED purging disorder.

f. Four individuals had OSFED purging disorder, one individual had OSFED bulimia nervosa and one individual had OSFED binge-eating disorder.

g. One individual with OSFED night eating syndrome.

*P < 0.05, **P < 0.01, ***P < 0.0001; anorexia nervosa *v.* comparison group.

### Full recovery from eating disorder symptoms

In the anorexia nervosa group, 64% (*n* = 30) were considered fully recovered. There was no significant difference in mean BMI between recovered and non-recovered participants (recovered: 22.7 kg/m^2^, s.d. 3.1; non-recovered: 24.5 kg/m^2^, s.d. 7.2; *P* = 0.24). The three individuals with current anorexia nervosa, including the two in partial remission, had a mean BMI of 19.7 kg/m^2^ (s.d. 3.1, range 16.3–22.4). Those with full recovery of eating disorder symptoms had significantly better outcome according to the GAF and Morgan–Russell averaged scale score than those without full eating disorder symptom recovery.

### Changes in eating disorder diagnoses between study 4 and study 5 and over 30 years

**Fig. 1:**
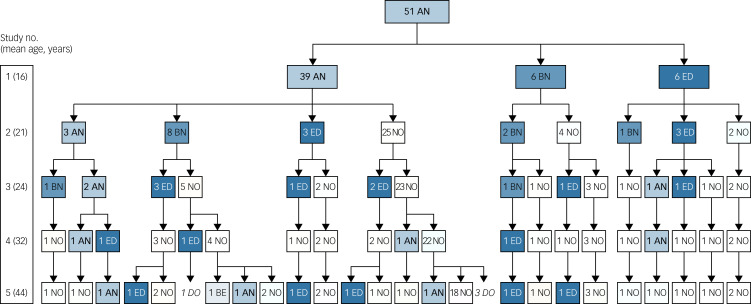
The trajectories of eating disorders over 30 years. The eating disorder diagnoses at each of the five assessments for all 51 individuals in the anorexia nervosa group is shown. The numbers before the abbreviations in the boxes correspond to the number of individuals with the condition. The column to the left shows the number of the study and, within brackets, the mean age of the anorexia nervosa group at the time of the study. From study 1 to study 4 the eating disorder diagnoses were assigned according to the DSM-IV; the DSM-5 criteria were applied at study 5. AN, anorexia nervosa; BE, binge-eating disorder; BN, bulimia nervosa; DO, dropped out; ED, eating disorder not otherwise specified (other specified feeding or eating disorder according to the DSM-5); NO, no eating disorder.

**Fig. 2:**
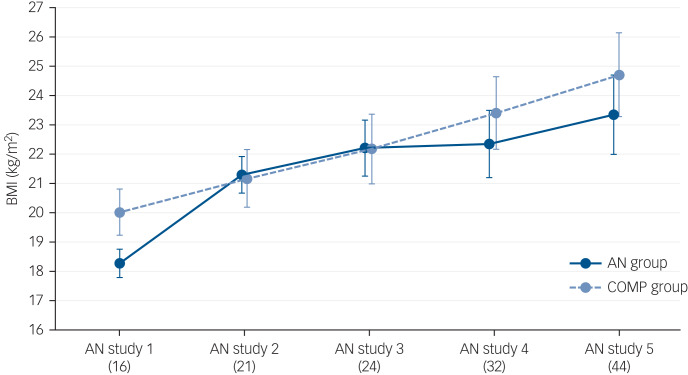
Body mass index (BMI) in the anorexia nervosa and comparison group in the original study and across the four follow-up studies. Average BMI in each group at each of the five assessments is displayed. The number below each study indicates the mean age of all participants at that assessment. Error bars indicate 95% CI. AN, anorexia nervosa; AN study 1, the original study; AN study 2, 6-year follow-up; AN study 3, 10-year follow-up; AN study 4, 18-year follow-up; AN study 5, 30-year follow-up (the present study); COMP, comparison.

Between the two most-recent follow-up studies, 17% (*n* = 8) of participants fulfilled the criteria for anorexia nervosa at some point and 32% (*n* = 15) had experienced any type of eating disorder (including anorexia nervosa) (Table 2; Supplementary Figure S1). Figure 1 shows the trajectories of the individuals' eating disorders over 30 years. All of those who had anorexia nervosa at study 4 were now free from any eating disorder. Figure 2 shows BMI over 30 years.

### Other psychiatric disorders at study 5

Psychiatric morbidity was significantly over-represented in the anorexia nervosa group (Table 2). DSM-5 anxiety disorders were the most common psychiatric disorders in both groups, but they were significantly more common in the anorexia nervosa group (Table 2).

### Changes in other psychiatric disorders between study 4 and study 5 and over 30 years

Between the two most-recent follow-up studies, psychiatric diagnoses were significantly more common in the anorexia nervosa group than in the comparison group (Table 2). The percentages of psychiatric disorders in the original study and across the four follow-up studies are available in Figure S2.

### Treatment received

In the anorexia nervosa group, 23% had never received treatment for an eating disorder. Only two individuals were in current treatment for an eating disorder at study 5, including one person with anorexia nervosa who received compulsory treatment. According to the mean Morgan–Russell averaged scale score and the mean GAF there was no difference between those who had ever received treatment for an eating disorder and those who had not received treatment.

### HRQoL

A total of 37 people from the anorexia nervosa group and 47 from the comparison group completed the SF-36 (Table S2). A drop-out analysis showed that people in the anorexia nervosa group who completed the survey had a significantly lower mean GAF score (58.3) than those who did not (69.8; *P* = 0.043), and psychiatric comorbidity was more common in those who completed the survey than in those that did not (*P* = 0.015). The MCS was significantly lower in the anorexia nervosa group (Supplementary Table S2). Neither the mental subscale scores nor the MCS were significantly lower among those in the anorexia nervosa group with a current eating disorder. Individuals in the anorexia nervosa group with a current psychiatric morbidity scored significantly lower in all subscale and composite scores than individuals without a psychiatric disorder.

### Correlations between previously diagnosed ASD and other variables

Our four previous studies have shown that 12% (*n* = 6) of the anorexia nervosa group had ASD at all four examinations (ASDx4).^6^ At study 5, the mean GAF and Morgan–Russell averaged scale score were significantly lower in the ASDx4 group compared with the remainder of the anorexia nervosa group. No individuals in the ASDx4 group had a current eating disorder at study 5.

### Predictive factors for good outcome

Stepwise linear regression analysis revealed that higher age at onset of adolescent-onset anorexia nervosa and perfectionism before onset of anorexia nervosa were individual predictors for better outcome on GAF, Morgan–Russell averaged scale score and MCS. Additionally, early gastrointestinal problems were an individual predictor for better outcome on the Morgan–Russell averaged scale score. In the stepwise logistic regression for full recovery from eating disorder symptoms, higher age at onset of adolescent-onset anorexia nervosa was a significant predictor (Tables S3 and S4).

## Discussion

This unique, controlled, 30-year follow-up study of adolescent-onset anorexia nervosa has shown that the majority of people with this disorder do well in the long-term perspective. There was no mortality, almost two-thirds reported full recovery from eating disorder symptoms and physical aspects of quality of life were similar across the anorexia nervosa and comparison groups. However, eating disorders were still present in a minority, affecting one in five.

Due to the sample being partly population based and including only cases of adolescent-onset anorexia nervosa, we had hypothesised that the outcome of our anorexia nervosa group would be better than other very long-term outcome studies. With no deaths in our sample, we had better mortality results than clinical outcome studies. Our findings were in line with the community-based FinnTwin study, which reported no mortality after 10 years.^21^ The proportion of full recovery from eating disorder symptoms (64% in our anorexia nervosa group) was not better than that seen in clinical studies with follow-up periods of more than 20 years.^8–10^

One other study has an observational period of more than 30 years.^10^ The sample had been admitted to hospital during the period 1931–1960. Given that no evidence-based treatment for anorexia nervosa had been developed during that period, it is surprising that 76% of the sample had recovered after 33 years. However, the mortality rate of 18% may be an indication of the lack of knowledge about eating disorders in health services at the time. Only 6% of participants had an eating disorder after 33 years.^10^ In our 18-year follow-up, we found 12% with an eating disorder.^6^ We expected to find, if anything, lower rates at the 30-year follow-up, but instead we saw a small increase. We were surprised to find that a third of the anorexia nervosa group fulfilled an eating disorder diagnosis during the past 12 years. However, this is in line with a recent 20-year follow-up study of in-patients with anorexia nervosa that showed a remission rate of only 39%. In that study, remission was defined as not fulfilling any eating disorder diagnosis (including ‘eating disorder syndrome below the threshold of DSM-IV’) for the past 3 months.^22^

The prospective design enabled us to study the individual trajectories of anorexia nervosa and crossover from one eating disorder to another. Between the 18- and 30-year follow-up, one in five had an eating disorder relapse. Diagnostic crossover involving anorexia nervosa, binge-eating disorder and other specified feeding or eating disorder was also common during this follow-up period. At the 10-year follow-up, study 3,^5^ half of the anorexia nervosa group had met criteria for bulimia nervosa at some point, which is in line with other long-term follow-up studies.^20^ However, from study 4 and onwards bulimia nervosa was no longer observed among the individuals in the anorexia nervosa group.

In this study, one in four people had never received treatment for an eating disorder. Nonetheless, treatment did not affect the outcome 30 years after the onset of anorexia nervosa. However, since the individuals were not randomly allocated to receive or not receive treatment, these outcome results must be interpreted with caution. Our finding is in accordance with a 5-year follow-up study of eating disorders where treatment did not affect the outcome of any eating disorder.^23^ Our results may reflect that individuals with anorexia nervosa are reluctant to undergo treatment and there is meagre scientific evidence for anorexia nervosa treatment *per se*, with least evidence for adult patients.^19^ The resistance to recovery posits anorexia nervosa among the most difficult psychiatric disorders to treat.

Regarding HRQoL, the PCS reflected a good outcome in the anorexia nervosa group, but the MCS did not. The lower MCS results could partly be explained by a significant over-representation of psychiatric morbidity and lower mean GAF among those who completed the SF-36 compared with those who did not. In our sample individuals with a current eating disorder did not express worse HRQoL compared with those who had no current eating disorder, whereas Eddy's group^9^ found the physical and psychological aspects of HRQoL to be significantly poorer among people with unrecovered anorexia nervosa at the 22-year follow-up. In a community-based study using the SF-36, individuals with a self-reported history of anorexia nervosa had a poorer HRQoL than those who did not have a history of anorexia nervosa.^24^

Later age at onset among individuals with adolescent-onset anorexia nervosa predicted good outcome, i.e. adolescent onset is better than childhood onset according to the GAF and the Morgan–Russell averaged scale score. This is consistent with outcome studies of childhood-onset anorexia nervosa,^25^ where the illness ‘often takes a chronic and disabling course with high morbidity rates’.^25^ The individuals in our study with the lowest ages of onset were in the same age range as the oldest children in the outcome studies of childhood-onset anorexia nervosa (which had an inclusion criterion of onset of anorexia nervosa before age 14).^25^ Premorbid perfectionism was a favourable prognostic factor even though premorbid perfectionism has often been reported as a risk factor for developing anorexia nervosa.^26^ Clinical perfectionism may maintain the eating disorder psychopathology^27^ and therefore an eating disorder treatment designed to produce enduring change, enhanced cognitive–behavioural therapy, has included a module for reducing clinical perfectionism. In contrast, perfectionism has been shown to persist in individuals with good outcome and anorexia nervosa recovery.^28^ Could it be that the perfectionism that drove the illness was diverted to driving recovery, i.e. perfectionism can both help and hinder in attaining a goal? Early severe gastrointestinal problems also predicted a good outcome in the present study. In our original study, M.R. found that half the individuals in the anorexia nervosa group had an early history of severe gastrointestinal problems.^7^ These symptoms may reflect an immature gastrointestinal tract that caused a great deal of concern in childhood and during adolescence and therefore contributed to the development of anorexia nervosa, but was subsequently not a risk factor for perpetuating anorexia nervosa.

### Strengths and limitations

To our knowledge, this is the only study that has followed people with adolescent-onset anorexia nervosa and matched comparison participants prospectively for 30 years. The sample is community based and half of the participants with anorexia nervosa constitute a total birth cohort. However, some factors may have influenced the course of the illness. Even if all individuals were initially identified in the community, half the group included a greater proportion of individuals who had been in contact with treatment facilities. The majority of individuals received treatment at some point, as opposed to the FinnTwin study where only half of the participants had been detected in the healthcare system.^3^ Even though some individuals in our anorexia nervosa group did not seek treatment, one must bear in mind that identifying and following the individuals prospectively for 30 years could be considered an intervention.

The comparison group was matched for age, gender and school, and has been followed since the original study. Only two other research groups have included a comparison group in their follow-up studies of anorexia nervosa.^29,30^ The drop-out rate after 30 years was extremely low; 96% agreed to examination for the fifth time in this project. The prospective design made it possible to study each individual's eating disorder trajectory in detail over a period of 30 years.

One of the weaknesses of this study pertains to the sample size: 47 individuals with adolescent-onset anorexia nervosa is a relatively small number compared with other very long-term studies where 84–121^8–10^ patients were followed up. However, the present study is the only one that can claim representativeness, with its population-/community-based design.

Individuals who decline participation in a follow-up study may represent those with the worst outcome.^9^ Our drop-out rate was 4%, corresponding to four individuals of the anorexia nervosa group. We had at least some follow-up information on all non-participants; we had been in touch with all of them and at least half of them worked full time. Based on these data we do not believe that our small group of non-participants represents those with the worst outcome.

Our definition of recovery (full recovery from eating disorder symptoms for a minimum of 6 consecutive months) could be questioned and it may have been better to use a longer time period. Some researchers have argued for a 1-year period without eating disorder behaviours, due to the risk of relapse being greatest within 1 year post-treatment or post-recovery.^20,31^ Our definition of recovery includes physical (BMI), behavioural (absence of binge eating, compensatory behaviours and restrictive eating) and psychological (body image concerns and fear of weight gain) aspects. Khalsa *et al*^31^ have reported that an instrument measuring psychological symptoms of eating disorders, e.g. the Eating Disorder Examination Questionnaire (EDE-Q),^32^ should be included in the assessment, and that individuals who exhibit full eating disorder recovery should score in accordance with community norms. A weakness of our study is that we did not use the EDE-Q as an outcome measure and therefore the more detailed aspects of eating disorder symptoms were not assessed. Each of the four other anorexia nervosa studies with a follow-up period of 20 years or more^8–10,22^ have used their own definition of recovery, with one research group arguing that their more strict definition resulted in a recovery rate of only 39%.^22^ According to a recent review of the definition of recovery of anorexia nervosa, Khalsa *et al*^31^ conclude that ‘to date there are no consensus guidelines available’ for research purposes.

Another limitation is that referral to eating disorder specialist treatment by the researchers during the 30-year follow-up period may have affected the outcomes of the study. At study 2, only a total of 37% of the population-based group had received treatment.^33^ At study 3, study 4 and study 5 the individuals with current anorexia nervosa did not agree to be referred to an eating disorder service.

### Clinical implications

Adolescent-onset anorexia nervosa carries a good ‘lifetime’ prognosis in terms of mortality and anorexia nervosa chronicity. However, high prevalence of anorexia nervosa relapses between the 18-year and the 30-year follow-ups indicates that late relapses occur, even though some individuals had been free from an eating disorder for one or two decades. As clinicians, we must be aware that a substantial minority of patients will continue to need psychiatric expertise for their eating disorder or other psychiatric disorders for many years.

Higher age at onset of adolescent-onset anorexia nervosa predicted better general outcome. This finding implies that – among school health nurses, school doctors, child psychiatrists and paediatricians – more effort needs to be made to detect individuals with a very early onset of anorexia nervosa, i.e. in childhood and early adolescence. With regard to the good outcomes predicted by premorbid perfectionism in this study, premorbid and clinical perfectionism in anorexia nervosa has traditionally been regarded as challenging by clinicians in terms of treatment outcome and prognosis. Clinically we may have to reconsider this dogma, at least in terms of the premorbid traits. However, premorbid perfectionism has to our knowledge not been reported as a good prognostic factor previously and therefore our finding needs to be replicated before taking further clinical actions.

## Supplementary material

For supplementary material accompanying this paper visit https://doi.org/10.1192/bjp.2019.113. click here to view supplementary material
