# Supplementary material for: Anorexia nervosa: 30-year outcome
Source: Br J Psychiatry. 2019 May 22;216(2):97–104. doi: 10.1192/bjp.2019.113 (PMC7557598; doi:10.1192/bjp.2019.113)
Supplement: Supplementary file 1 [file S0007125019001132sup001.zip › S0007125019001132sup001.docx]

Table S4. Logistic regression model for predictive factors of full ED symptom recovery

|  | B (SE) | OR (95% CI) | Wald χ^2^ (df=1) | p | Cox & Snell R^2^ |
| --- | --- | --- | --- | --- | --- |
| Model 1 |  |  |  |  |  |
| Age at AN onset | 0.82 (0.38) | 2.26 (1.08, 4.72) | 4.71 | 0.030 | .34 |
| Perfectionism | 2.02 (1.27) | 7.52 (0.62, 91.41) | 2.51 | 0.113 |  |
| Premorbid BMI | -0.03 (0.13) | 0.97 (0.75, 1.26) | 0.05 | 0.829 |  |
| Perinatal factors | -0.46 (0.30) | 0.63 (0.35, 1.13) | 2.40 | 0.121 |  |
|  |  |  |  |  |  |
| Model 2 |  |  |  |  |  |
| Age at AN onset | 0.83 (0.38) | 2.30 (1.10, 4.80) | 4.91 | 0.027 | .34 |
| Perfectionism | 2.06 (1.26) | 7.82 (0.66, 92.63) | 2.66 | 0.103 |  |
| Premorbid BMI | -0.48 (0.28) | 0.62 (0.36, 1.07) | 2.96 | 0.086 |  |
|  |  |  |  |  |  |
| Model 3 |  |  |  |  |  |
| Age at AN onset | 0.77 (0.34) | 2.17 (1.12, 4.21) | 5.21 | 0.022 | .28 |
| Perfectionism | -0.41 (0.24) | 0.67 (0.41, 1.07) | 2.79 | 0.095 |  |
|  |  |  |  |  |  |

*Note*. ED: eating disorder; AN: anorexia nervosa; BMI: body mass index; BSTEP (Backward stepwise) procedure in SPSS was used (removal criterion: p > 0.10, entry criterion p < 0.50), Model 2 vs. Model 1: Δχ^2^ = 0.46, p = 0.830, Model 3 vs. Model 2: Δ χ^2^ = 3.45, p = 0.063.
