# Supplementary material for: Anorexia nervosa: 30-year outcome
Source: Br J Psychiatry. 2019 May 22;216(2):97–104. doi: 10.1192/bjp.2019.113 (PMC7557598; doi:10.1192/bjp.2019.113)
Supplement: Supplementary file 1 [file S0007125019001132sup001.zip › S0007125019001132sup004.docx]

|  | **AN (n=47)** | | | | **COMP (n=51)** | | | | **p** |
| --- | --- | --- | --- | --- | --- | --- | --- | --- | --- |
|  | Mean | SD | Median | Range | Mean | SD | Median | Range |  |
| **Ages  AN Study 5** | 44.42 | 1.84 | 44.78 | 38.47-47.57 | 44.22 | 1.77 | 44.46 | 38.44-46.74 | 0.58 |
| **Follow-up period between  AN onset and  *AN Study 5*** | 30.13 | 1.62 | 30.01 | 27.11-33.87 | N.A. |  |  |  |  |
| **Follow-up period between  *AN Study 4* and  *AN Study 5*** | 12.02 | 0.57 | 12.06 | 10.39-13.11 | 11.84 | 0.53 | 11.89 | 10.56-12.76 | 0.10 |
| **Duration  *AN Study 1* to  *AN Study 5*** | 28.25 | 2.15 | 28.68 | 22.35-32.97 | 28.15 | 2.31 | 28.58 | 21.14-34.24 | 0.82 |

**Table S1. Ages and follow-up duration (years) at *AN Study 5* of the AN and COMP group, respectively**

AN: anorexia nervosa group; COMP: comparison group; *AN Study 1*: The original study; *AN Study 4*: 18-year follow-up; *AN Study 5*: 30-year follow-up, the present study; SD: standard deviation; N.A.: not applicable
