# Supplementary material for: Anorexia nervosa: 30-year outcome
Source: Br J Psychiatry. 2019 May 22;216(2):97–104. doi: 10.1192/bjp.2019.113 (PMC7557598; doi:10.1192/bjp.2019.113)
Supplement: Supplementary file 1 [file S0007125019001132sup001.zip › S0007125019001132sup005.docx]

**Table S2. Health-related quality of life (SF-36) scores in the AN and COMP group at**

***AN Study 5***

| **HRQoL subscales** | **AN group N=37** | | **COMP group N=47** | | **p** |
| --- | --- | --- | --- | --- | --- |
|  | **score** | **SD** | **score** | **SD** |  |
| **Physical health components** |  |  |  |  |  |
| Physical functioning | 55.01 | 4.06 | 55.63 | 4.65 | 0.523 |
| Role physical | 51.73^a^ | 9.40 | 52.82^b^ | 7.32 | 0.560 |
| Bodily pain | 51.95 | 10.24 | 54.06^c^ | 9.37 | 0.332 |
| General health | 52.24 | 13.39 | 56.13 | 9.96 | 0.130 |
| Physical health  summary | 54.75^a^ | 7.34 | 54.88^b^ | 7.35 | 0.937 |
| **Mental health components** |  |  |  |  |  |
| Vitality | 47.48^a^ | 11.85 | 51.41^b^ | 9.47 | 0.101 |
| Social functioning | 48.53 | 12.02 | 52.22 | 7.20 | 0.085 |
| Role emotional | 48.91^a^ | 10.80 | 52.69^b^ | 6.68 | 0.057 |
| Mental health | 46.07^a^ | 12.67 | 52.44^b^ | 7.41 | 0.006 |
| Mental health summary | 45.02^a^ | 13.44 | 51.22^b^ | 7.83 | 0.011 |

SF-36: The short form health survey; *AN Study 5*: 30-year follow-up, the present study; AN: anorexia nervosa; COMP: comparison; HRQoL: Health-related quality of life; SD: standard deviation; ^a^ based on 36 individuals; ^b^ based on 45 individuals; ^c^ based on 46 individuals
