# Supplementary material for: Anorexia nervosa: 30-year outcome
Source: Br J Psychiatry. 2019 May 22;216(2):97–104. doi: 10.1192/bjp.2019.113 (PMC7557598; doi:10.1192/bjp.2019.113)
Supplement: Supplementary file 1 [file S0007125019001132sup001.zip › S0007125019001132sup006.pdf]

Table S3. Linear regression models for predictive factors of good outcome

	B (95% CI)	SE	β	t	p	R^2
Global assessment of functioning (GAF)						
Age at AN onset	3.98 (1.33, 6.63)	1.31	0.38	3.03	0.004	0.32
Perfectionism	17.54 (7.45, 27.62)	5.00	0.44	3.51	0.001	
Morgan-Russell averaged scale score						
Model 1						
Age at AN onset	0.33 (0.00, 0.66)	0.16	0.29	2.06	0.047	0.36
Perfectionism	1.44 (0.18, 2.70)	0.62	0.32	2.32	0.027	
Early GI problems	0.99 (-0.04, 2.03)	0.51	0.27	1.95	0.060	
Perinatal factors	-0.21 (-0.53, 0.11)	0.16	-0.19	-1.35	0.185	
Model 2						
Age at AN onset	0.37 (0.04, 0.70)	0.16	0.32	2.30	0.028	0.32
Perfectionism	1.41 (0.14, 2.69)	0.63	0.31	2.25	0.031	
Early GI problems	1.05 (0.00, 2.09)	0.51	0.29	2.03	0.050	
SF-36 Mental Composite Score (MCS)						
Model 1						
Age at AN onset	3.72 (1.46, 5.98)	1.11	0.48	3.35	0.002	0.35
Perfectionism	8.98 (-0.56, 18.52)	4.68	0.28	1.92	0.064	
Early GI problems	3.42 (-4.67, 11.52)	3.98	0.13	0.86	0.395	
Model 2						
Age at AN onset	3.85 (1.62, 6.08)	1.10	0.50	3.52	0.001	0.33
Perfectionism	9.92 (0.69, 19.15)	4.54	0.31	2.19	0.036	
SF-36 Physical Composite Score (PCS)						
Perfectionism	4.79 (-1.04, 10.62)	2.87	0.28	1.67	0.104	0.08

Note. AN: anorexia nervosa; GI: gastrointestinal; BACKWARD procedure in SPSS was used (removal criterion: $p > 0.10$, entry criterion $p < 0.50$), Morgan-Russell averaged scale score Model 2 vs. Model 1: $\Delta F = 1.83$, $p = 0.185$, SF-36 Mental Composite Score Model 2 vs. Model 1: $\Delta F = 0.74$, $p = 0.395$. GI = gastrointestinal.
