# Supplementary figures and images for: Anorexia nervosa: 30-year outcome
Source: Br J Psychiatry. 2019 May 22;216(2):97–104. doi: 10.1192/bjp.2019.113 (PMC7557598; doi:10.1192/bjp.2019.113)

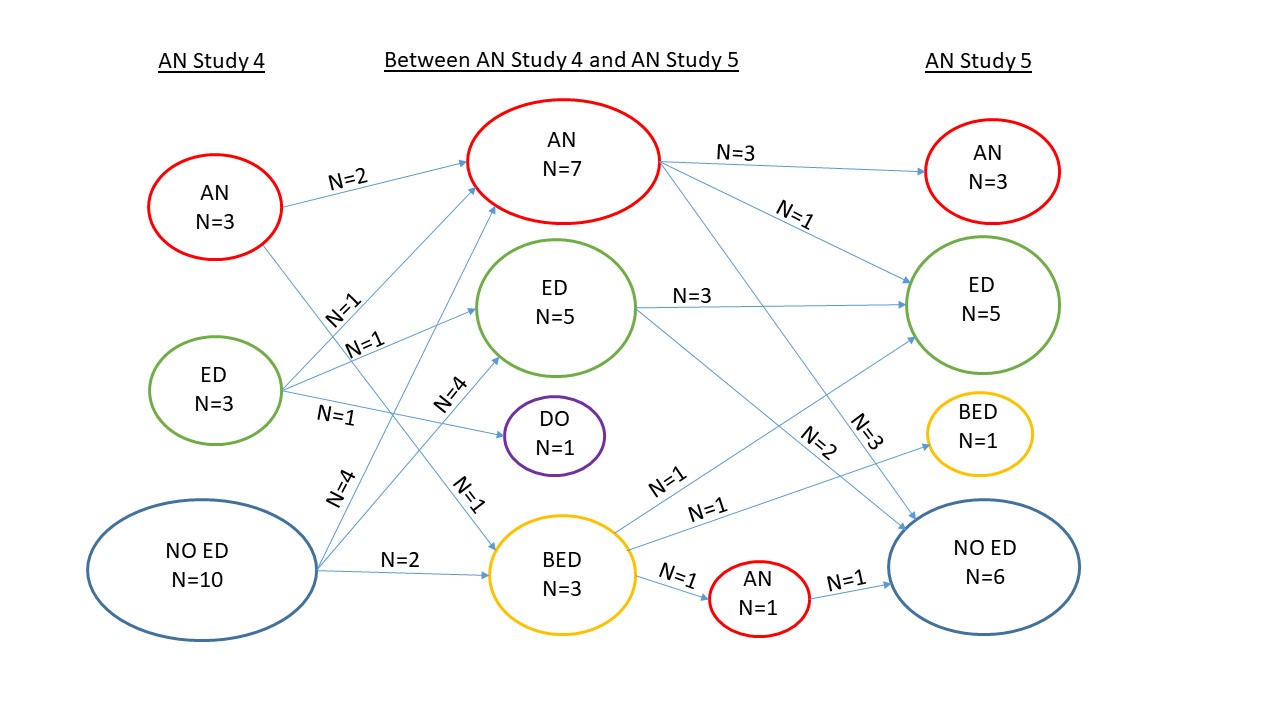

Supplement: Supplementary file 1 [file S0007125019001132sup001.zip › S0007125019001132sup002.jpg]

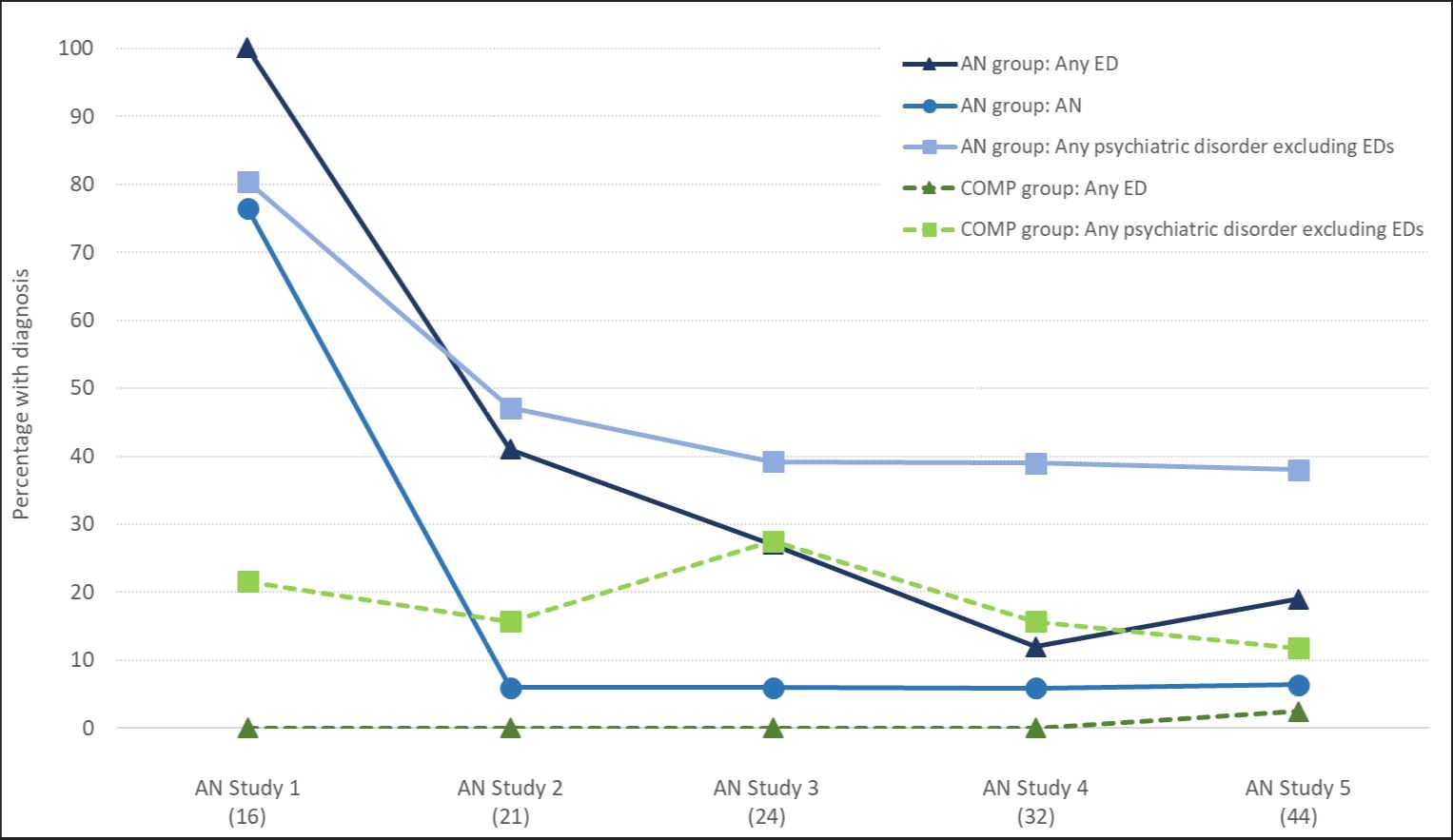

Supplement: Supplementary file 1 [file S0007125019001132sup001.zip › S0007125019001132sup003.jpg]
